# Endoscopic ultrasound-guided drainage using a forward-viewing echoendoscope for peripancreatic fluid collection after Child resection

**DOI:** 10.1055/a-2234-4075

**Published:** 2024-01-30

**Authors:** Takuya Ishikawa, Kentaro Yamao, Yasuyuki Mizutani, Tadashi Iida, Kota Uetsuki, Masanao Nakamura, Hiroki Kawashima

**Affiliations:** 1 36589 Department of Gastroenterology and Hepatology, Nagoya University Graduate School of Medicine Faculty of Medicine, Nagoya, Japan; 2 36590 Department of Endoscopy, Nagoya University Hospital, Nagoya, Japan; 3 36589 Gastroenterology and Hepatology, Nagoya University Graduate School of Medicine Faculty of Medicine, Nagoya, Japan; 4 36589 Department of Gastroenterology and Hepatology, Nagoya University Graduate School of Medicine Faculty of Medicine, Nagoya, Japan

Peripancreatic fluid collection (PFC) due to pancreatic leakage is one of the significant complications arising following pancreatic surgery. Although endoscopic ultrasound (EUS)-guided drainage is less invasive than other methods in treating PFCs, it requires the drainage area to be in contact with the gastrointestinal tract accessible by the echoendoscope 1 . In patients with surgically altered anatomy, an oblique-viewing echoendoscope, which is mainly used for EUS-guided drainage, encounters technical difficulties in the jejunum. Recently, the usefulness of a forward-viewing echoendoscope in such cases has been reported 2 3 .

We experienced a case of afferent limb syndrome due to postoperative PFC at the pancreaticojejunal anastomosis following a modified Child resection in a 70-year-old man ( Fig. 1 ). As the fluid collection was remotely located from the stomach ( Fig. 2 ), and the afferent limb was in close proximity ( Fig. 3 ), EUS-guided drainage was attempted using a forward-viewing echoendoscope (TGF-UC260J; Olympus Optical, Tokyo, Japan) ( Video 1 ). Confirming the direction to reach the afferent limb was made relatively easy using the forward-viewing echoendoscope. Based on computed tomography (CT) findings, the target fluid collection could be confirmed by EUS observation where the scope was withdrawn a few centimeters from the choledochojejunal anastomosis. A 19G needle was used for puncture, a guidewire was placed, and the puncture site was dilated using a 4-mm tapered balloon. For double-wire indwelling, a double-lumen catheter was used, and the procedure was completed with placement of both internal and external stents. Immediately following the procedure, the patient showed symptom improvement, and 2 months later CT confirmed complete disappearance of the PFC ( Fig. 4 ).

**Fig. 1:**
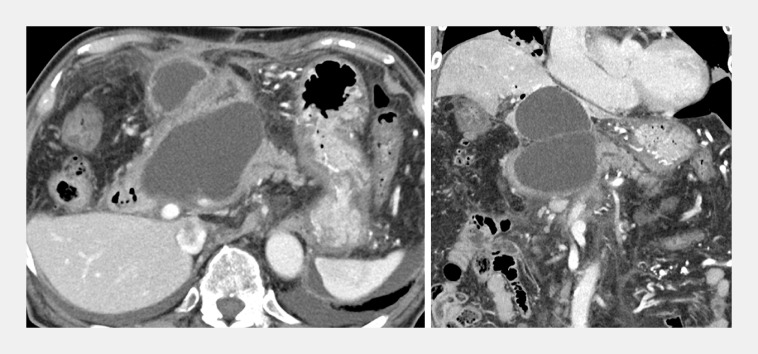
Computed tomography (CT) showing a large pancreatic fluid collection at the pancreaticojejunal anastomosis site following modified Child resection in a 70-year-old man.

**Fig. 2:**
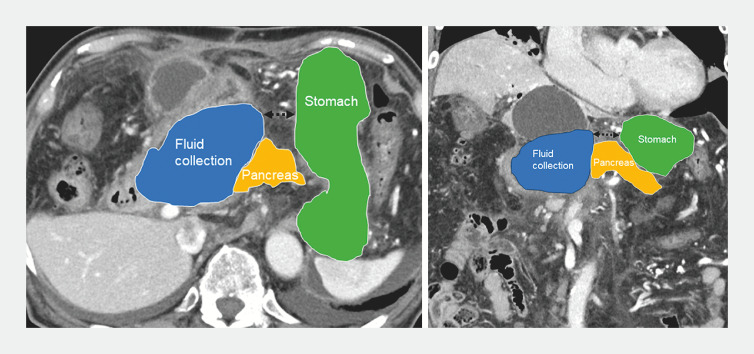
CT with overlaid schema showing the distance between the fluid collection and the stomach. As the fluid collection developed at the pancreaticojejunal anastomosis site, it was remotely located from the stomach.

**Fig. 3:**
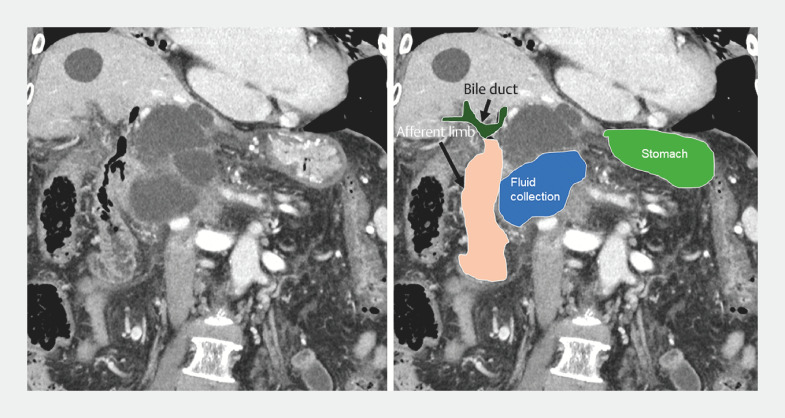
CT and the same image with overlaid schema showing the afferent limb and the fluid collection in close proximity.

**Fig. 4:**
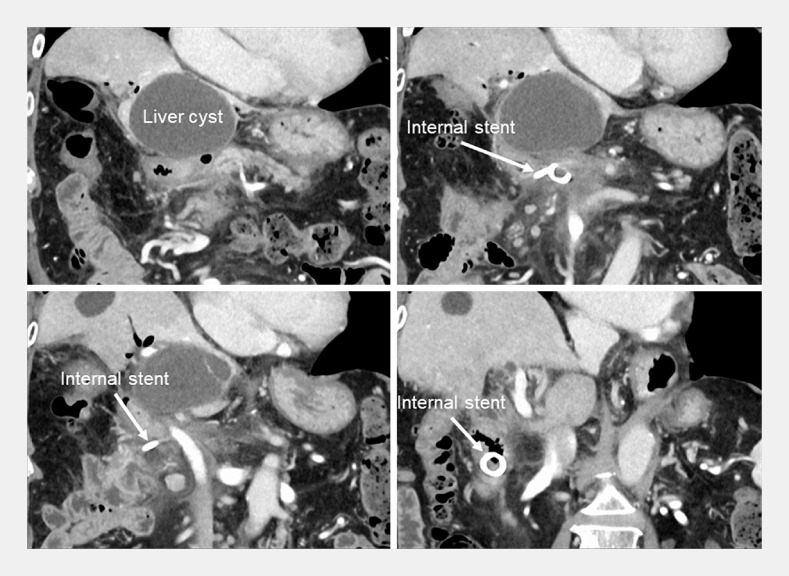
CT taken 2 months following drainage showing complete disappearance of the fluid collection.

Endoscopic ultrasound-guided drainage using a forward-viewing echoendoscope for peripancreatic fluid collection following modified Child resection.Video 1

Conventional EUS-guided drainage predominantly employs an oblique-viewing echoendoscope, which presents considerable challenges and perforation risks when intubating in the afferent limb. In contrast, the forward-viewing echoendoscope provides a superior and safer approach, offering precise echo-guided puncture determination.

Endoscopy_UCTN_Code_TTT_1AS_2AD

## Notes

Endoscopy E-Videos https://eref.thieme.de/e-videos
*E-Videos* is an open access online section of the journal *Endoscopy* , reporting on interesting cases and new techniques in gastroenterological endoscopy. All papers include a high-quality video and are published with a Creative Commons CC-BY license. Endoscopy E-Videos qualify for HINARI discounts and waivers and eligibility is automatically checked during the submission process. We grant 100% waivers to articles whose corresponding authors are based in Group A countries and 50% waivers to those who are based in Group B countries as classified by Research4Life (see: https://www.research4life.org/access/eligibility/ ). This section has its own submission website at https://mc.manuscriptcentral.com/e-videos .
